# Support for evidence-based alcohol policy in Ireland: results from a representative household survey

**DOI:** 10.1093/eurpub/ckad031

**Published:** 2023-03-02

**Authors:** Susan Calnan, Seán R Millar, Deirdre Mongan

**Affiliations:** School of Public Health, University College Cork, Cork, Ireland; School of Public Health, University College Cork, Cork, Ireland; Health Research Board, Dublin, Ireland

## Abstract

**Background:**

Alcohol use is a leading risk factor for death and disability and there is a need for evidence-based policy measures to tackle excess alcohol consumption and related harms. The aim of this study was to examine attitudes towards alcohol control measures among the general public in the context of significant reforms undertaken in the Irish alcohol policymaking landscape.

**Methods:**

A representative household survey was conducted among individuals aged 18+ years in Ireland. Descriptive and univariate analyses were used.

**Results:**

A total of 1069 participants took part (48% male) and there was broad support (>50%) for evidence-based alcohol policies. Support was strongest for a ban on alcohol advertising near schools and creches (85.1%) and for warning labels (81.9%). Women were more likely than men to support alcohol control policy measures while participants with harmful alcohol use patterns were significantly less likely to support these measures. Respondents with a greater awareness of the health risks of alcohol showed higher levels of support, while those who had experienced harms due to other people’s drinking showed lower support compared with those who had not experienced such harms.

**Conclusions:**

This study provides evidence of support for alcohol control policies in Ireland. However, notable differences were found in levels of support according to sociodemographic characteristics, alcohol consumption patterns, knowledge of health risks and harms experienced. Further research on reasons behind public support towards alcohol control measures would be worthwhile, given the importance of public opinion in the development of alcohol policy.

## Introduction

Alcohol use is a leading risk factor for death and disability globally and is linked to 60 acute and chronic diseases.^1^^,^^2^ While alcohol is deeply embedded in the social landscape of many countries and is linked to sociability and friendship,^3^^,^^4^ the significant health, social and economic impacts of alcohol outweigh such benefits. Some 3 million deaths worldwide were attributed to alcohol use in 2016 and 132.6 million disability-adjusted life years (DALYs).^3^ Mortality from alcohol consumption is higher than that from diseases such as tuberculosis, HIV/AIDS and diabetes.^5^ Alcohol use also adversely affects 14 of the 17 United Nations Sustainable Development Goals and is considered an obstacle to all three dimensions (social, environmental and economic) of sustainable development.^6^

The European Union (EU) is the region with the highest alcohol consumption in the world, and alcohol is the third leading risk factor for disease and mortality in Europe.^7^ Within the EU, Ireland has one of the highest per capita alcohol consumption rates, with alcohol consumption levels forecast to increase over the next decade in this country.^8^ Moreover, most drinkers in Ireland continue to consume alcohol in a manner that is risky to their health. Data from the Healthy Ireland Survey 2016 indicate that more than half (52.3%) of Irish drinkers were classified as hazardous drinkers using the World Health Organization’s (WHO) AUDIT-C screening tool. Almost two-fifths (39.2%) of drinkers engaged in monthly heavy episodic drinking (HED) and one-fifth (22.8%) engaged in HED on a weekly basis.^9^

The significant and persistent toll of alcohol on society reinforces the need for evidence-based policy measures to tackle alcohol consumption and related harms. Such measures are now a common feature of legal and regulatory systems throughout the world.^10^ In Ireland, there has been a shift from a liberalized to a more regulated approach to alcohol consumption, most notably through enactment of the Public Health (Alcohol) Act in 2018.^11^ Hailed as ‘a world-leading package of policy reforms’ in its capacity to address ‘the cross-cutting nature of the policy problems’,^12^ the Act comprises a suite of policy measures including minimum unit pricing, structural separation of alcohol in mixed retail outlets, restrictions on alcohol advertising and marketing and health labelling requirements for alcohol products.^11^ These measures align with the ‘best buy’ policy measures recommended by the WHO to help reduce harmful alcohol use.^13^

Policy implementation is an under-researched but growing field as governments and policy stakeholders recognize the importance of the policy implementation process and that legislative enactment does not automatically guarantee policy success.^14^ One aspect of implementation is in relation to public attitudes to, and support for, policy reforms. Negative public attitudes to policies may lead to problems with implementation and adherence.^15^ Public support can also be an influence on political decision-making in terms of which policies are supported by governments;^16^ studies suggest that public opinion might be the single most important explanation for alcohol policy.^17^ International research also indicates that public opinion appears to be divided regarding alcohol control measures,^18^ with strong support for less intrusive lighter touch policies (e.g. education and information campaigns) but less support for policies addressing the price and availability of alcohol.^19^

Previous research has found support for evidence-based alcohol control policy in Ireland.^20^ However, now that implementation of these policy measures is underway, it is important to determine if this support has been sustained and continue to gauge public attitudes to alcohol policy measures. Therefore, the aim of this study was to examine attitudes towards alcohol control measures among the general public in the context of the significant reforms underway in the Irish alcohol policymaking landscape. In particular, we examined sociodemographic characteristics, alcohol consumption patterns, knowledge of health risks and experience of alcohol-related harms that may influence such attitudes.

## Methods

### Study setting

This study was conducted in southern Ireland in three areas where the National Community Action on Alcohol Pilot Project (NCAAPP) is underway, an initiative aimed at supporting regional drug and alcohol taskforces to adopt a ‘community mobilization’ approach to reducing alcohol-related harms.^21^ The project aligns with national and international policy aimed at promoting community action to address alcohol-related harms,^22^^,^^23^ including the WHO Global Action Plan 2022–30 to Strengthen Implementation of the Global Strategy to Reduce the Harmful Use of Alcohol.^3^ The three sites comprise one urban area in a large city and two towns on opposite points of the alcohol strategy group’s region. Local drug and alcohol taskforces support the work of the pilot sites, each of which has access to community workers, meeting spaces and steering group members.

### Survey and sampling

A household survey was undertaken by a reputable market research company among individuals living in southern Ireland. Participants were members of the general population, aged 18 and over, living in the three NCAAPP pilot areas. To achieve a representative sample of residents within each specific area, a quota-based approach was employed, with quotas set to reflect the population of that specific area. This involved a two-stage sampling approach. Firstly, a stratified random selection of geographical points; and secondly a selection of respondents within geographical points (to meet specified quotas). These quotas were set to reflect sex, age and working status to match the population (aged 18 years and over) of that area in line with the most recent Irish census data (2016). Funding for the study was provided by the Cork Local Drug and Alcohol Taskforce, who were collaborators and knowledge users for this study. Ethical approval to conduct the research was granted by the Social Research Ethics Committee at University College Cork.

### Data collection

Data collection was undertaken from July to August 2022. Trained interviewers conducted a face-to-face questionnaire with sampled participants. In addition to sociodemographic information, questions focused on alcohol consumption, health awareness and harm, and were based on a survey questionnaire used in a previous study, albeit with some additions.^20^ Alcohol consumption was investigated using the WHO’s Alcohol Use Disorders Identification Test (AUDIT), a validated set of 10 questions designed to examine a person’s alcohol use.^24^ The AUDIT-C uses the first three questions of the AUDIT questionnaire. In this study, hazardous drinking was defined as an AUDIT-C score of 4 or more among males and 3 or more among females.^25^^,^^26^ These scoring criteria are recommended based on previous validation studies.^25^^,^^27^^,^^28^ The lower recommended cut-point in women reflects the lower threshold for risky drinking in women and the fact that women often under-report alcohol consumption more than men, potentially because of greater stigma.^25^ Full AUDIT-10 scores were also calculated for survey participants, with scores of 0–7 indicating ‘low risk’ drinking, 8–15 indicating ‘increased risk’, 16–19 indicating ‘high risk’ and with scores ≥20 suggesting alcohol dependence.^24^

### Data analysis

Trained researchers coded, entered and cleaned the data. Data were then weighted for sex and age in line with population figures from Ireland’s 2016 Census. We undertook descriptive and univariate analyses to investigate sociodemographic, alcohol consumption pattern, health risk knowledge and alcohol-related harm relationships with support for evidence-based alcohol policy measures. Data analysis was conducted using Stata SE Version 13 (Stata Corporation, College Station, TX, USA) for Windows. For all analyses, a *P* values (two-tailed) of less than 0.05 was considered to indicate statistical significance.

## Results

### Descriptive characteristics

A total of 1069 participants took part in the survey (48% male, 52% female). Full demographic results are shown in table 1.

**Table 1:** Sociodemographic information and alcohol consumption patterns of survey respondents—full sample and stratified by sex

Variable	All subjects	Males	Females	*P*
Age category				
18–24	124 (11.6)	70 (13.5)	54 (9.8)	0.339
25–34	158 (14.9)	68 (13.2)	91 (16.5)	
35–44	203 (19.0)	99 (19.1)	104 (18.9)	
45–54	186 (17.5)	90 (17.4)	96 (17.5)	
55–64	152 (14.3)	70 (13.5)	82 (14.9)	
65+	243 (22.8)	120 (23.2)	123 (22.4)	
Education				
Primary only	126 (11.9)	70 (13.6)	57 (10.3)	0.082
Junior certificate	193 (18.1)	91 (17.6)	102 (18.5)	
Leaving certificate	370 (34.7)	192 (37.2)	178 (32.3)	
Diploma	212 (19.9)	89 (17.2)	123 (22.3)	
Primary or postgraduate degree	163 (15.3)	74 (14.3)	90 (16.3)	
Refusal	1 (0.1)	0 (0.0)	1 (0.2)	
Marital status				
Single	307 (28.8)	163 (31.6)	145 (26.4)	<0.001
Cohabiting	144 (13.5)	69 (13.4)	75 (13.6)	
Married	405 (38.0)	205 (39.7)	199 (36.2)	
Divorced or separated	104 (9.7)	48 (9.3)	56 (10.2)	
Widowed	102 (9.6)	28 (5.4)	74 (13.5)	
Refusal	4 (0.4)	3 (0.6)	1 (0.2)	
Employment				
Working for pay or self-employed	460 (43.2)	250 (48.5)	210 (38.1)	<0.001
Involuntary/voluntary unemployed	84 (7.9)	49 (9.5)	36 (6.5)	
Student	74 (6.9)	37 (7.2)	37 (6.7)	
Retired	255 (23.9)	138 (26.8)	116 (21.1)	
Illness	65 (6.1)	38 (7.4)	27 (4.9)	
Homemaker	128 (12.0)	3 (0.6)	125 (22.7)	
AUDIT-C				
Non-hazardous drinking	512 (48.2)	221 (43.0)	291 (53.1)	0.001
Hazardous drinking	550 (51.8)	293 (57.0)	257 (46.9)	
AUDIT-10				
Low risk	848 (79.8)	354 (68.9)	494 (90.1)	<0.001
Increased risk	171 (16.1)	128 (24.9)	43 (7.8)	
High risk	17 (1.6)	14 (2.7)	3 (0.5)	
Alcohol dependent	26 (2.5)	18 (3.5)	8 (1.5)	
Binge drinking				
Never	278 (35.3)	88 (23.0)	189 (47.1)	<.0001
Less than monthly	230 (29.3)	109 (28.5)	121 (30.2)	
Monthly	111 (14.1)	62 (16.2)	49 (12.2)	
Weekly	143 (18.2)	105 (27.4)	37 (9.2)	
Daily/almost daily	25 (3.1)	19 (5.0)	5 (1.2)	
Drinking to intoxication				
Less than weekly	695 (87.9)	315 (81.6)	380 (93.8)	<0.001
Weekly	96 (12.1)	71 (18.4)	25 (6.2)	

Notes: Displayed frequencies and percentages (in parentheses) are weighted. Numbers may not add up in column totals because of missing data. *P* for sex differences determined using a χ^2^ test.

Regarding alcohol consumption, 74.5% of participants reported that they drink alcohol (the remaining 25.5% of the sample stated that they ‘never’ consume alcohol). Of those who consumed alcohol, 51.8% of participants were in the hazardous drinking category based on the AUDIT-C scoring. A higher proportion of males (57%) than females (46.9%) were in this category. With regard to AUDIT-10 score categories, 3.5 and 1.5% of males and females, respectively, were classified as being alcohol dependent. Over one-third (35.4%) of respondents reported binge drinking on a monthly or more frequent (weekly or daily) basis. In addition, over one-tenth (12.1%) reported drinking to intoxication on a weekly basis, with nearly three times as many men than women falling into this category.

**Figure 1.**
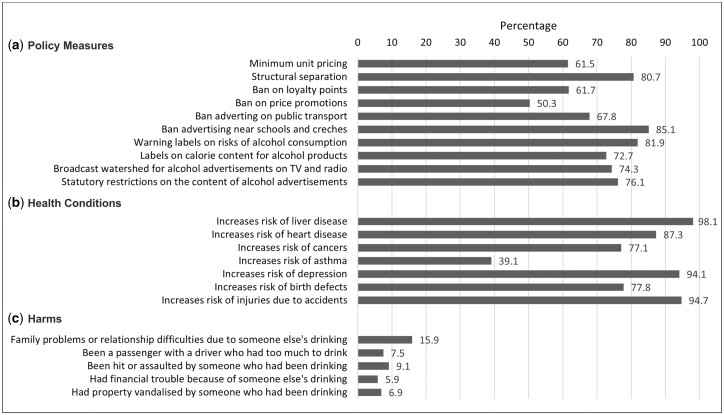
(a–c) Percentage of respondents showing support for alcohol policy measures, awareness of negative health impacts and experience of alcohol-related harms.

There was broad support (>50%) among participants for evidence-based alcohol policies legislated under the Public Health (Alcohol) Act (figure 1a). Support was strongest for a ban on alcohol advertising near schools and creches (85.1%) and for warning labels on alcohol products (81.9%). A ban on price promotions and support for minimum unit pricing garnered the lowest levels of support overall at 50.3 and 61.5%, respectively. Levels of opposition (% disagree) were also highest for these two measures (Supplementary table S1) at 33.8 and 26.9%, respectively. The percentage of ‘don’t knows’ was highest for a ban on advertising on public transport and a ban on loyalty points at 18.7 and 18%, respectively. A majority showed awareness of the potential negative health impacts of alcohol—particularly, in relation to liver disease, depression and risk of injury due to accidents (>90%) (figure 1b). Almost 16% of participants had experienced family problems or relationship difficulties due to someone else’s drinking while 9.1% had been hit or assaulted by someone who had been drinking (figure 1c).

### Support for evidence-based alcohol policies

Women were more likely than men to support alcohol policy measures (table 2). Significant differences between women and men were found in relation to support for the following policy measures: statutory restrictions on the content of alcohol advertisements (80.4 vs. 71.5%; *P *=* *0.001); minimum unit pricing (66.2 vs. 56.4%; *P *=* *0.001); a ban on alcohol advertising near schools and creches (87.5 vs. 82.6%; *P *=* *0.025); warning labels on risks of alcohol (84.4 vs. 79.5%; *P *=* *0.037); structural separation of alcohol products (83.6 vs. 77.7%; *P *=* *0.014); a broadcast watershed for alcohol advertisements on TV and radio (77.3 vs. 71.1%; *P *=* *0.022); and a ban on alcohol advertising on public transport (70.7 vs. 64.7%; *P *=* *0.036). Participants who were married or widowed expressed the strongest support for alcohol policy measures compared with other marital status groups. The lowest level of support in this category was found among participants who were separated or divorced.

**Table 2:** Percentage of survey respondents who supported evidence-based alcohol policy measures in relation to sociodemographic information and alcohol consumption patterns

Variable	Minimum unit pricing	Structural separation in mixed retail outlets	Ban on loyalty points	Ban on price promotions	Ban adverting on public transport	Ban advertising near schools and creches	Warning labels on risks of alcohol consumption	Labels on calorie content for alcohol products	Broadcast watershed for alcohol advertisements on TV and radio	Statutory restrictions on the content of alcohol advertisements
Sex										
Male	291 (56.4)	401 (77.7)	303 (58.8)	247 (47.9)	334 (64.7)	426 (82.6)	410 (79.5)	357 (69.2)	367 (71.1)	369 (71.5)
Female	364 (66.2)	460 (83.6)	354 (64.4)	289 (52.5)	389 (70.7)	481 (87.5)	464 (84.4)	418 (76.0)	425 (77.3)	442 (80.4)
Age category										
18–24	56 (45.2)	102 (82.3)	74 (59.7)	39 (31.5)	74 (59.7)	106 (85.5)	101 (81.5)	93 (75.0)	92 (74.2)	100 (80.6)
25–34	85 (53.8)	121 (76.1)	85 (53.5)	59 (37.1)	99 (62.3)	134 (84.8)	128 (81.0)	123 (77.8)	110 (69.6)	114 (72.2)
35–44	116 (57.4)	163 (80.7)	122 (60.4)	95 (46.8)	136 (67.3)	176 (87.1)	165 (81.7)	153 (75.7)	153 (75.7)	144 (71.3)
45–54	115 (61.8)	145 (78.0)	113 (60.8)	100 (53.8)	122 (65.6)	154 (82.8)	147 (78.6)	124 (66.7)	128 (68.4)	131 (70.4)
55–64	106 (69.3)	123 (80.9)	104 (68.4)	94 (61.8)	112 (73.2)	128 (84.2)	129 (84.9)	102 (67.1)	112 (73.7)	121 (79.6)
65+	177 (73.1)	206 (84.8)	159 (65.7)	149 (61.3)	180 (74.1)	209 (86.0)	202 (83.5)	179 (73.7)	197 (91.4)	200 (82.3)
Education										
Primary only	80 (63.5)	92 (73.0)	72 (56.7)	70 (55.6)	80 (63.5)	91 (71.7)	102 (80.3)	88 (69.3)	84 (66.7)	90 (70.9)
Junior certificate	117 (60.6)	145 (75.1)	109 (56.5)	98 (50.8)	131 (67.9)	166 (86.0)	158 (81.9)	132 (68.4)	136 (70.5)	142 (73.6)
Leaving certificate	229 (61.9)	307 (82.7)	226 (61.1)	187 (50.4)	266 (71.7)	328 (88.6)	301 (81.4)	267 (72.2)	275 (74.1)	292 (78.9)
Diploma	125 (59.0)	177 (83.5)	143 (67.5)	102 (48.1)	134 (63.2)	176 (83.0)	174 (82.1)	162 (76.4)	163 (76.9)	156 (73.6)
Primary or postgraduate degree	104 (63.8)	140 (85,4)	108 (65.9)	79 (48.5)	111 (68.1)	147 (89.6)	137 (84.0)	126 (76.8)	134 (82.2)	130 (79.8)
Marital status										
Single	163 (52.9)	247 (80.5)	177 (57.7)	129 (41.9)	191 (62.2)	262 (85.3)	244 (79.5	220 (71.7)	226 (73.4)	232 (75.6)
Cohabiting	83 (57.6)	113 (78.5)	84 (57.9)	55 (38.2)	96 (66.7)	123 (85.4)	117 (81.2)	111 (77.1)	104 (72.2)	104 (72.2)
Married	288 (71.1)	347 (85.9)	281 (69.4)	244 (60.4)	298 (73.6)	353 (87.4)	338 (83.5)	300 (74.3)	315 (78.0)	323 (79.8)
Divorced or separated	44 (42.3)	64 (61.5)	47 (45.6)	41 (39.8)	58 (56.3)	76 (73.1)	82 (78.8)	61 (58.7)	60 (57.7)	62 (59.6)
Widowed	76 (74.5)	87 (85.3)	68 (66.7)	65 (63.7)	77 (75.5)	90 (88.2)	89 (87.3)	80 (78.4)	84 (82.4)	87 (85.3)
Employment										
Working for pay or self-employed	267 (57.9)	368 (80.0)	275 (59.7)	210 (45.6)	309 (67.0)	387 (83.9)	377 (82.0)	344 (74.6)	340 (73.9)	337 (73.3)
Involuntary/voluntary unemployed	47 (55.3)	65 (76.5)	44 (52.4)	41 (48.2)	52 (61.9)	68 (81.0)	68 (80.0)	52 (61.2)	59 (70.2)	58 (68.2)
Student	32 (43.2)	58 (78.4)	47 (63.5)	25 (33.8)	44 (59.5)	66 (89.2)	58 (78.4)	50 (67.6)	54 (73.0)	63 (85.1)
Retired	185 (72.5)	212 (83.1)	165 (64.7)	155 (60.8)	183 (71.8)	216 (85.0)	210 (82.7)	185 (72.5)	204 (80.0)	207 (81.5)
Illness	37 (57.8)	55 (84.6)	43 (66.2)	35 (54.7)	47 (72.3)	59 (90.8)	55 (84.6)	47 (73.4)	44 (68.8)	46 (70.8)
Homemaker	87 (68.0)	103 (80.5)	85 (66.4)	71 (55.5)	88 (68.8)	111 (86.7)	106 (82.8)	96 (75.0)	90 (70.9)	99 (78.0)
AUDIT-C										
Non-hazardous drinking	386 (75.4)	445 (86.9)	365 (71.3)	335 (65.4)	407 (79.5)	465 (90.8)	461 (89.9)	399 (77.9)	427 (83.2)	439 (85.7)
Hazardous drinking	268 (48.7)	414 (75.3)	291 (52.9)	200 (36.3)	315 (57.2)	441 (80.2)	410 (74.5)	373 (67.8)	363 (66.0)	369 (67.1)
AUDIT-10										
Low risk	588 (69.3)	721 (85.0)	556 (65.6)	482 (56.8)	622 (73.3)	748 (88.2)	731 (86.2)	650 (76.7)	677 (79.8)	700 (82.5)
Increased risk	59 (34.5)	125 (73.1)	90 (52.6)	48 (28.1)	88 (51.5)	135 (79.4)	109 (63.7)	103 (60.2)	98 (57.3)	97 (56.7)
High risk	6 (33.3)	10 (58.8)	6 (33.3)	3 (17.6)	9 (52.9)	13 (76.5)	14 (82.4)	11 (64.7)	11 (64.7)	7 (41.2)
Alcohol dependent	2 (7.7)	3 (11.5)	5 (19.2)	1 (3.8)	3 (11.1)	10 (37.0)	16 (61.5)	8 (29.6)	3 (11.5)	4 (15.4)
Binge drinking										
Never	191 (69.0)	224 (80.9)	183 (66.1)	152 (54.7)	189 (68.0)	238 (85.9)	240 (86.6)	222 (80.1)	223 (80.2)	221 (79.8)
Less than monthly	141 (61.3)	208 (90.8)	154 (67.0)	110 (48.0)	162 (70.7)	208 (90.4)	190 (82.6)	178 (77.4)	179 (77.8)	193 (84.3)
Monthly	54 (49.1)	85 (77.3)	59 (53.6)	38 (34.2)	68 (61.3)	91 (82.7)	78 (70.9)	74 (67.3)	78 (70.3)	77 (70.0)
Weekly	48 (33.8)	89 (62.7)	63 (44.4)	37 (26.1)	64 (44.8)	105 (73.4)	98 (68.5)	85 (59.4)	72 (50.7)	71 (49.7)
Daily/almost daily	3 (12.0)	10 (41.7)	2 (8.0)	1 (4.0)	5 (20.0)	13 (52.0)	12 (48.0)	6 (24.0)	7 (28.0)	5 (20.0)
Drinking to intoxication										
Less than weekly	417 (59.9)	573 (82.4)	431 (61.9)	316 (45.5)	453 (65.1)	598 (86.0)	561 (80.7)	518 (74.4)	510 (73.4)	525 (75.4)
Weekly	26 (27.1)	51 (53.1)	36 (37.9)	28 (29.2)	41 (42.7)	63 (65.6)	63 (65.6)	53 (55.8)	53 (55.2)	49 (51.6)

Notes: Displayed frequencies and percentages (in parentheses) are weighted.

Younger age groups (18–24 years) were less likely than older age groups to support minimum unit pricing, a ban on price promotions and a ban on alcohol advertising on public transport. Those with the lowest level of education (some primary) were less likely to support a ban on alcohol advertising near schools and creches as well as a broadcast watershed on alcohol advertising. Regarding employment status, students were less likely to support minimum unit pricing and a ban on price promotions, while self-employed people were less likely to support structural separation of alcohol products, a broadcast watershed and statutory restrictions on content of alcohol advertising.

Participants with hazardous or harmful alcohol use patterns were significantly less likely to support evidence-based alcohol policy measures compared with their low-risk drinking counterparts. Based on the AUDIT-C scoring, for example, there was a significant difference (*P *<* *0.001) in support between hazardous and non-hazardous drinkers across all policy measures, particularly in relation to support for a ban on price promotions (36.3 vs. 65.4%), minimum unit pricing (48.7 vs. 75.4%), and a ban on alcohol advertising on public transport (57.2 vs. 79.5%). A significant difference (*P *<* *0.001) in support was similarly observed for the AUDIT-10 categories, with support for alcohol policy measures generally waning by the severity of consumption. Survey respondents who refrained from binge drinking were also significantly more likely (*P *<* *0.001) to support alcohol policy measures than those who reported binge drinking on a monthly or more frequent (weekly or daily) basis. The same was true when comparing those who indicated drinking to intoxication on a less than weekly basis and those who did so on a weekly basis.

In relation to levels of support for policy measures according to knowledge of health risks, participants who had a greater awareness of the health risks associated with alcohol were more likely to support evidence-based policy measures (table 3). For example, significant differences in levels of support were found between those aware of the cancer-related risks of alcohol vs. those unaware of the risks, particularly regarding support for calorie content labelling on alcohol products (76.5 vs. 59.7%), a ban on alcohol advertising on public transport (71.4 vs. 55.3%) and a broadcast watershed on alcohol advertisements (77.5 vs. 63.5%) (*P *<* *0.001 for all).

**Table 3:** Percentage of survey respondents who supported evidence-based alcohol policy measures in relation to those who agreed that consumption of alcoholic beverages increases the risk of health conditions

Variable	Minimum unit pricing	Structural separation in mixed retail outlets	Ban on loyalty points	Ban on price promotions	Ban adverting on public transport	Ban advertising near schools and creches	Warning labels on risks of alcohol consumption	Labels on calorie content for alcohol products	Broadcast watershed for alcohol advertisements on TV and radio	Statutory restrictions on the content of alcohol advertisements
Increases risk of liver disease										
No	7 (35.0)	12 (60.0)	10 (50.0)	8 (40.0)	14 (70.0)	13 (65.0)	14 (66.7)	12 (60.0)	14 (66.7)	10 (50.0)
Yes	648 (62.0)	848 (81.1)	647 (61.9)	527 (50.4)	709 (67.8)	895 (85.6)	860 (82.2)	762 (72.8)	778 (74.4)	800 (76.6)
Increases risk of heart disease										
No	68 (50.4)	92 (68.1)	74 (54.8)	53 (39.3)	75 (55.1)	98 (72.6)	99 (73.3)	86 (63.2)	83 (61.5)	76 (56.3)
Yes	587 (63.1)	768 (82.6)	583 (62.7)	482 (51.8)	648 (69.6)	809 (87.0)	774 (83.1)	689 (74.1)	709 (76.2)	734 (78.9)
Increases risk of cancers										
No	129 (52.9)	191 (78.6)	143 (58.6)	100 (41.2)	135 (55.3)	190 (77.9)	181 (74.2)	145 (59.7)	155 (63.5)	160 (65.6)
Yes	526 (64.0)	669 (81.3)	514 (62.5)	435 (52.9)	587 (71.4)	718 (87.3)	693 (84.2)	629 (76.5)	637 (77.5)	651 (79.1)
Increases risk of asthma										
No	376 (57.9)	506 (78.0)	372 (57.3)	298 (45.9)	391 (60.2)	527 (81.2)	509 (78.4)	439 (67.7)	456 (70.4)	455 (70.1)
Yes	280 (67.0)	355 (84.9)	286 (68.4)	238 (57.1)	332 (79.6)	381 (91.1)	364 (87.3)	335 (80.3)	336 (80.4)	356 (85.4)
Increases risk of depression										
No	30 (47.6)	38 (60.3)	33 (52.4)	28 (44.4)	36 (57.1)	45 (71.4)	43 (68.3)	38 (60.3)	37 (58.7)	36 (57.1)
Yes	625 (62.3)	822 (82.0)	624 (62.2)	508 (50.6)	687 (68.5)	863 (86.0)	830 (82.8)	736 (73.4)	755 (75.3)	775 (77.2)
Increases risk of birth defects										
No	126 (53.4)	164 (69.2)	133 (56.1)	93 (39.2)	116 (49.2)	170 (71.7)	183 (77.2)	150 (63.3)	138 (58.2)	148 (62.4)
Yes	529 (63.7)	696 (84.0)	525 (63.3)	443 (53.4)	607 (73.1)	738 (88.9)	691 (83.3)	625 (75.3)	654 (78.9)	663 (79.9)
Increases risk of injuries due to accidents										
No	28 (50.0)	33 (57.9)	29 (50.9)	29 (50.9)	31 (54.4)	40 (70.2)	38 (66.7)	28 (49.1)	36 (63.2)	34 (59.6)
Yes	627 (62.1)	827 (82.0)	629 (62.3)	507 (50.2)	692 (68.5)	867 (85.9)	835 (82.8)	746 (73.9)	756 (74.9)	777 (77.0)

Notes: Displayed frequencies and percentages (in parentheses) are weighted.

Surprisingly, participants who had experienced harms due to other people’s drinking showed lower support for alcohol control policies compared with those who had not experienced such harms (Supplementary table S2). To investigate this, we conducted sensitivity analyses; it was found that survey respondents who had experienced such alcohol-related harms were also more likely to have engaged in harmful alcohol consumption patterns themselves (Supplementary table S3). For instance, nearly twice as many hazardous drinkers than non-hazardous drinkers had experienced family or relationship problems due to someone else’s drinking (20.9 vs. 10.7%) and four times as many hazardous than non-hazardous drinkers had been a passenger with a driver who had too much to drink (11.6 vs. 2.9%) (*P *<* *0.001 for both).

## Discussion

### Principal findings

In this study, we examined attitudes towards evidence-based alcohol control policies using a representative household survey in Ireland. Findings from this research suggest broad support for alcohol control policies among the Irish population. However, levels of support differed according to sociodemographic characteristics, alcohol consumption patterns, knowledge of health risks and harms experienced. Levels of alcohol consumption remain high, with over half of those surveyed falling into the hazardous drinking category and over one-third reporting binge drinking on a monthly or more frequent (weekly or daily) basis.

### Interpretation of our findings

#### Support for alcohol policies by demographics and type of policy

This study demonstrates considerable support for measures of the recently enacted Public Health (Alcohol) Act, with over 50% of participants expressing support for measures contained in the Act. These findings correlate with previous research, which showed support for government measures targeting alcohol consumption among the Irish population at a community level.^20^^,^^29^ However, similar to previous research conducted in Ireland and elsewhere (England, Scotland and Australia), women, older age groups and low-risk drinkers were more likely to support alcohol control policies.^16^^,^^20^^,^^30^^,^^31^ Such findings underline Karlsson et al.’s observation that while public support seems to have shifted in favour of more restrictive and less liberal positions in recent decades, public opinion is not necessarily unified.^32^

This study also found differences in levels of support according to type of alcohol control policy measure, with the strongest support (>80%) being found for a ban on alcohol advertising near schools and creches and for warning labels on alcohol products, while price restrictions generated the lowest level of support and the highest level of opposition (i.e. a ban on price promotions and minimum unit pricing). Once again, this complements research conducted in Ireland and internationally, which suggests that public support may wane as policy measures become more restrictive.^16^^,^^20^^,^^32^ It also underlines the somewhat paradoxical relationship between public support and policy effectiveness, whereby public support for policies to reduce alcohol consumption and harms have been found to be inversely associated with policy effectiveness; for instance, policies with the greatest evidence for effectiveness, such as pricing and availability, are often the least popular.^33^ While this is possibly due to the fact that financial considerations (i.e. the cost of consumer products) may outweigh concerns regarding the health impacts of alcohol, it reinforces the need for greater awareness raising among the general public regarding the effectiveness of price increases, as a greater understanding of the rationale behind alcohol control policy measures may increase public support for such measures.^34^ For instance, it is noteworthy that the proportion of ‘don’t knows’ was among the highest regarding support for a ban on loyalty points for alcohol products. This suggests that some views on policy measures may be due to a lack of understanding rather than outright opposition to such measures, further underlining the need for more proactive strategies to raise awareness regarding the rationale and effectiveness of alcohol policy measures among the general public.

Among the policy measures outlined in this study, support for warning labels on alcohol products was one of the highest, at 81.9% overall. A growing body of research indicates public support for alcohol labelling,^35^^,^^36^ including a recent opinion poll in Ireland which found overwhelming support among those surveyed for alcohol labelling on the health risks and calorific content of alcohol products.^37^ These are important findings given that alcohol labelling is among the provisions of Ireland’s Public Health (Alcohol) Act that remains unimplemented, and which has been delayed by alcohol industry intervention. As evidence grows regarding the potential positive impact of warning labels on alcohol products,^38–40^ the concomitant public support for such measures could be further leveraged to encourage the Irish government to proceed with implementing this policy measure. As Critchlow et al. conclude, strong political leadership is likely needed in order to meet consumers’ basic rights to be informed about the potential harms of alcohol products available for purchase.^41^

#### Support for policies by alcohol consumption levels, awareness of health risks and harms

A further finding from this study was the significantly lower levels of support for alcohol policy measures among those with hazardous or harmful alcohol use patterns compared with low-risk drinkers. Other studies similarly note lower levels of support for restrictive alcohol policies among those who drink more.^16^^,^^20^^,^^42^ While this is not necessarily surprising, further research on why this is the case would be worthwhile. One possible explanation for example, is that those who drink at higher levels may be more susceptible to alcohol industry framings which highlight the importance of ‘responsible drinking’—a ‘strategically ambiguous, industry-affiliated term’ that shifts the focus on individual responsibility with a view to ‘reducing the threat of regulation’.^43^ The finding that those with greater awareness of the health risks of alcohol were more likely to support evidence-based policy measures is also noteworthy. It may suggest, for instance, that greater awareness raising of the health risks of alcohol could also increase support for alcohol control measures—an important consideration for both policymakers and public health stakeholders.

One notable difference in this study compared with other research is the lower levels of support for alcohol policy measures among those who had experienced harms due to other people’s drinking. This contrasts with results from other studies, which suggest that experience of harm caused by other people’s drinking may increase support for more restrictive policies.^20^^,44^ However, closer analysis of data from our study may help clarify this observation—namely, that those who had experienced such harms were also more likely to engage in harmful drinking themselves (see Supplementary table S3). Since those who drink more are also more likely to support liberal policies,^16^^,^^32^^,42^ this offers a possible explanation for this finding. Moreover, it raises the possibility that in the heavier drinking categories, people’s own unhindered access to alcohol may trump any concerns over harms due to others’ drinking and a perceived need for alcohol control measures. Indeed, further exploration of this, for example through more in-depth qualitative research, could help to shed greater light on the possible reasons for differing attitudes between hazardous and non-hazardous drinkers regarding alcohol policy measures.

Understanding the reasons for public opinion on alcohol policies is complex and research is still only emerging on this topic. A recent study by Karlsson et al.^32^ in Sweden noted that narrow self-interest had some impact on public opinion in that frequent alcohol consumers most affected by restrictions were less likely to support restrictive policies. Importantly, however, the authors concluded that perceptions of the problematic societal consequences of alcohol, in combination with ideological norms regarding the responsibility of individuals, were far more important in explaining public opinion than self-interest factors. In particular, the view that there is a problem at the societal level, rather than at the personal level, was deemed most essential for explaining opinions on alcohol restrictions; personal experiences of close affiliates’ excessive drinking did not seem to influence the opinions expressed. Such conclusions may help to explain the finding in our study regarding lower support for policies among those experiencing harms due to other people’s drinking. The potential impact of the media on public support for alcohol policies is a further aspect worth exploring. For example, a study by Mercille^45^ on media coverage of alcohol issues in Ireland notes the media’s ‘clear reluctance to support strong public health strategies’. Since the news media are a primary source of information on public affairs for many people,^46^ it is possible that negative or a lack of media coverage may influence public attitudes to alcohol policies. Further research on reasons behind public opinion towards alcohol policies, including media influences, would be worthwhile in an Irish context therefore, given the importance of public opinion in the development of alcohol policy.^17^

### Strengths and limitations

A key strength of this study is the robust sampling strategy used to attain a representative sample of the population. Applying a household sampling strategy helped to ensure that the sample reflects the wider population in the respective areas, in line with the most recent Irish census data. One potential limitation is the risk of social desirability bias in face-to-face surveys, particularly in relation to sensitive topics such as drug or alcohol use.^47^ Use of skilled interviewers and moving questions of a more sensitive nature further down in the survey were among the measures taken to enhance the validity of responses.

## Conclusions

This study provides further evidence of support for alcohol control policies in Ireland, notably those contained in Ireland’s Public Health (Alcohol) Act. However, notable differences were found in levels of support according to type of policy, sociodemographic characteristics, alcohol consumption patterns, awareness of alcohol-related health risks and harms experienced. This underlines the reality that notwithstanding considerable support for control measures, such support is not necessarily unified. Continued research on public opinions regarding alcohol policy may help to shed further light on reasons for such differences. The high levels of hazardous alcohol consumption and binge drinking found in this study reinforces the need for full implementation of policy measures enacted in Ireland. Evaluation and monitoring of these policy measures’ implementation, including possible changes in public support, should remain a priority to help inform international research on factors influencing alcohol control policies.

## Supplementary Material

ckad031_Supplementary_Data

## Data Availability

The data used and analysed for the purpose of this study are available from the corresponding author on reasonable request. Key points There is broad public support for more restrictive and less liberal alcohol control policies, as contained in Ireland’s Public Health (Alcohol) Act. However, levels of support vary according to type of policy, sociodemographic characteristics, alcohol consumption patterns, awareness of alcohol-related health risks and harms experienced. Surprisingly, participants who had experienced harms due to other people’s drinking showed lower support for alcohol control policies compared with those who had not experienced such harms. This study underlines the somewhat paradoxical relationship between public support and policy effectiveness, whereby public support for policies to reduce alcohol consumption and harms have been found to be inversely associated with policy effectiveness, e.g. policies with the greatest evidence for effectiveness, such as pricing and availability, are often the least popular.

## References

[1] Griswold MG , Fullman N, Hawley C, et al Alcohol use and burden for 195 countries and territories, 1990–2016: a systematic analysis for the Global Burden of Disease Study 2016. Lancet 2018;392:1015–35. 30146330 10.1016/S0140-6736(18)31310-2 PMC6148333

[2] Rehm J , Room R, Graham K, et al The relationship of average volume of alcohol consumption and patterns of drinking to burden of disease: an overview. Addiction 2003;98:1209–28. 12930209 10.1046/j.1360-0443.2003.00467.x

[3] World Health Organization (WHO). Global Action Plan 2022-2030 to Strengthen Implementation of the Global Strategy to Reduce the Harmful Use of Alcohol. Geneva: WHO: 2021.

[4] Thurnell-Read T. Identity, friendship and sociality. The SAGE Handbook of Drug & Alcohol Studies: Social Science Approaches. London: Sage; 2016: 337–351.

[5] World Health Organization (WHO). Global Status Report on Alcohol and Health 2018. Geneva: WHO; 2018.

[6] Movendi International. Alcohol Use: Fuelling the NCDs Tsunami. Stockholm: Movendi International; 2020. https://movendi.ngo/wp-content/uploads/2020/02/Alcohol-and-NCDs.pdf

[7] World Health Organization (WHO). Alcohol in the European Union: Consumption, Harm and Policy Approaches. Copenhagen: WHO Regional Office for Europe; 2012.

[8] Manthey J , Shield KD, Rylett M, et al Global alcohol exposure between 1990 and 2017 and forecasts until 2030: a modelling study. Lancet 2019;393:2493–2502. 31076174 10.1016/S0140-6736(18)32744-2

[9] O’Dwyer C , Mongan D, Doyle A, Galvin B. Alcohol Consumption, Alcohol-related Harm and Alcohol Policy in Ireland. HRB Overview Series 11. Dublin: HRB; 2021.

[10] Babor T , Caetano R, Casswell S, et al Alcohol No Ordinary Commodity – Research and Public Policy, 2nd edn. Oxford: Oxford University Press; 2010.

[11] Government of Ireland. Public Health (Alcohol) Act 2018. Available at: http://www.irishstatutebook.ie/eli/2018/act/24/enacted/en/html (23 February 2023, date last accessed).

[12] Lesch M , McCambridge J. A long-brewing crisis: the historical antecedents of major alcohol policy change in Ireland. Drug Alcohol Rev 2022;41:135–43. 34085320 10.1111/dar.13331

[13] World Health Organization (WHO). Tackling NCDs: ‘Best Buys’ and Other Recommended Interventions for the Prevention and Control of Noncommunicable Diseases. Geneva: WHO; 2017.

[14] Hudson B , Hunter D, Peckham S. Policy failure and the policy-implementation gap: can policy support programs help? Policy Des Pract 2019;2:1.

[15] Kaskutas L. Differential perceptions of alcohol policy effectiveness. J Public Health Policy 1993;14:413. 8163633

[16] Li J , Lovatt M, Eadie D, et al Public attitudes towards alcohol control policies in Scotland and England: results from a mixed-methods study. Soc Sci Med 2017;177:177–89. 28171817 10.1016/j.socscimed.2017.01.037 PMC5341733

[17] Baggott R. Alcohol, politics and social policy. J Soc Pol 1986;15:467–88.

[18] Tobin C , Moodie AR, Livingstone C. A review of public opinion towards alcohol controls in Australia. BMC Public Health 2011;11:1–9. 21272368 10.1186/1471-2458-11-58 PMC3048532

[19] Room R , Babor T, Rehm J. Alcohol and public health. Lancet 2005;365:519–30. 15705462 10.1016/S0140-6736(05)17870-2

[20] Davoren MP , Lane D, Kirby J, et al Support for evidence-based alcohol policy in Ireland: results for the Community Action on Alcohol Pilot Project. J Public Health Pol 2019;40:76–90. 10.1057/s41271-018-0151-y 30382156

[21] Timony Meehan A , Lyons S. National Community Action on Alcohol Pilot Project. Drugnet Ireland, Dublin: HRB; 2015.

[22] Department of Health. Steering Group Report on a National Substance Misuse Strategy. Dublin: Department of Health; 2012.

[23] Department of Health. Healthy Ireland: A Framework for Improved Health and Well-Being 2013-2025. Dublin: Department of Health; 2013.

[24] Babor TF , Higgins-Biddle JC, Saunders JB, Monteiro MG. The Alcohol Use Disorders Identification Test: Guidelines for Use in Primary Care, 2nd edn. Geneva: World Health Organization; 2001.

[25] Bradley KA , DeBenedetti AF, Volk RJ, et al AUDIT-C as a brief screen for alcohol misuse in primary care. Alcoholism Clin Exp Res 2007;31:1208–17. 10.1111/j.1530-0277.2007.00403.x 17451397

[26] O’Connor EA , PerdueLA, SengerCA, et alScreening and behavioral counseling interventions to reduce unhealthy alcohol use in adolescents and adults: updated evidence report and systematic review for the US Preventive Services Task Force. JAMA2018;320:1910.3042219810.1001/jama.2018.12086

[27] Bush K , Kivlahan DR, McDonell MB, et al The AUDIT alcohol consumption questions (AUDIT-C): an effective brief screening test for problem drinking. Ambulatory Care Quality Improvement Project (ACQUIP). Alcohol Use Disorders Identification Test. Arch Intern Med 1998;158:1789–95. 9738608 10.1001/archinte.158.16.1789

[28] Bradley KA , Bush KR, Epler AJ, et al Two brief alcohol-screening tests from the Alcohol Use Disorders Identification Test (AUDIT): validation in a female Veterans Affairs patient population. Arch Intern Med 2003;163:821. 12695273 10.1001/archinte.163.7.821

[29] Butler S. Ireland’s Public Health (Alcohol) Bill policy window or political sop. Contemp Drug Problems 2015;42:106–17.

[30] Tobin C , Moodie AR, Livingstone C. A review of public opinion towards alcohol controls in Australia. BMC Public Health 2011;11:58. 21272368 10.1186/1471-2458-11-58 PMC3048532

[31] Wilkinson C , Room R, Livingston M. Mapping Australian public opinion on alcohol policies in the new millennium. Drug Alcohol Rev 2009;28:263–74. 21462409 10.1111/j.1465-3362.2009.00027.x

[32] Karlsson D , Holmberg S, Weibull L. Solidarity or self-interest? Public opinion in relation to alcohol policies in Sweden. Nordic Stud Alcohol Drugs 2020;37:105–21. 10.1177/1455072520904644 PMC7434170 32934597

[33] Buykx P , Li J, De Matos EG, et al Factors associated with public support for alcohol policy in England: a population-based survey. Lancet 2016;388:S31.

[34] Storvoll EE , Rossow I, Rise J. Changes in attitudes towards restrictive alcohol policy measures: the mediating role of changes in beliefs. J Substance Use 2014;19:38–43. 24719564 10.3109/14659891.2012.728671 PMC3971770

[35] Martin-Moreno JM , Harris ME, Breda J, et al Enhanced labelling on alcoholic drinks: reviewing the evidence to guide alcohol policy. Eur J Public Health 2013;23:1082–87. 23657783 10.1093/eurpub/ckt046

[36] Wilkinson C , Room R. Warnings on alcohol containers and advertisements: international experience and evidence on effects. Drug Alcohol Rev 2009;28:426–35. 19594797 10.1111/j.1465-3362.2009.00055.x

[37] Alcohol Action Ireland (AAI). New poll reveals strong public support for health warnings in alcohol labelling, on-product. Alcohol Action Ireland [online]. Poll conducted by Ireland Thinks on behalf of AAI, 2022. Available at: https://alcoholireland.ie/new-poll-reveals-strong-public-support-for-health-warnings-in-alcohol-labelling-on-product/# (23 February 2023, date last accessed).

[38] Zhao J , Stockwell T, Vallance K, et al The effects of alcohol warning labels on population alcohol consumption: an interrupted time series analysis of alcohol sales in Yukon. Can J Stud Alcohol Drugs 2020;81:225–37. 32359054

[39] Jones D , Moodie C, Purves RI, et al Health information, messaging, and warnings on alcohol packaging: a focus group study with young adult drinkers in Scotland. Addict Res Theory 2021;29:469–78.

[40] Additional references 40–47 are shown in the Supplementary file.

