# Supplementary material for: Support for evidence-based alcohol policy in Ireland: results from a representative household survey
Source: Eur J Public Health. 2023 Mar 2;33(2):323–30. doi: 10.1093/eurpub/ckad031 (PMC10066492; doi:10.1093/eurpub/ckad031)
Supplement: ckad031_Supplementary_Data [file ckad031_supplementary_data.docx]

**Supplementary File**

**Table S1.** *Levels of support for evidence-based alcohol policy measures (% agree, disagree, don’t knows)*

| **Policy Measure** | **Agree (%)** | **Disagree (%)** | **Neutral/don’t know (%)** |
| --- | --- | --- | --- |
| Minimum unit pricing | 61.5 | 26.9 | 11.7 |
| Structural separation | 80.7 | 8.0 | 11.3 |
| Ban on loyalty points | 61.7 | 20.4 | 18.0 |
| Ban on price promotions | 50.3 | 33.8 | 15.9 |
| Ban adverting on public transport | 67.8 | 13.6 | 18.7 |
| Ban advertising near schools and creches | 85.1 | 4.0 | 10.9 |
| Warning labels on risks of alcohol consumption | 81.9 | 8.7 | 9.4 |
| Labels on calorie content for alcohol products | 72.7 | 14.0 | 13.3 |
| Broadcast watershed for alcohol advertisements on TV and radio | 74.3 | 9.0 | 16.7 |
| Statutory restrictions on the content of alcohol advertisements | 76.1 | 7.7 | 16.2 |

Displayed percentages are weighted.

**Table S2.** *Percentage of survey respondents who supported evidence-based alcohol policy measures in relation to those who had experienced harms due to someone else’s drinking*

| **Variable** | **Minimum unit pricing** | **Structural separation in mixed retail outlets** | **Ban on loyalty points** | **Ban on price promotions** | **Ban adverting on public transport** | **Ban advertising near schools and creches** | **Warning labels on risks of alcohol consumption** | **Labels on calorie content for alcohol products** | **Broadcast watershed for alcohol advertisements on TV and radio** | **Statutory restrictions on the content of alcohol advertisements** |
| --- | --- | --- | --- | --- | --- | --- | --- | --- | --- | --- |
| **Family problems or relationship difficulties due to someone else's drinking** |  |  |  |  |  |  |  |  |  |  |
| No | 570 (63.6) | 736 (82.1) | 555 (61.9) | 470 (52.5) | 622 (69.4) | 773 (86.3) | 729 (81.4) | 659 (73.5) | 681 (76.0) | 696 (77.7) |
| Yes | 86 (50.6) | 124 (72.9) | 102 (60.0) | 66 (38.8) | 101 (59.4) | 134 (78.8) | 144 (84.7) | 115 (67.6) | 111 (65.3) | 115 (67.6) |
| **Been a passenger with a driver who had too much to drink** |  |  |  |  |  |  |  |  |  |  |
| No | 630 (63.9) | 818 (83.0) | 620 (62.9) | 515 (52.2) | 685 (69.4) | 849 (86.1) | 811 (82.2) | 727 (73.7) | 752 (76.2) | 766 (77.7) |
| Yes | 25 (31.6) | 42 (52.5) | 37 (46.2) | 21 (26.2) | 38 (47.5) | 58 (72.5) | 62 (78.5) | 47 (59.5) | 40 (50.6) | 45 (56.2) |
| **Been hit or assaulted by someone who had been drinking** |  |  |  |  |  |  |  |  |  |  |
| No | 618 (63.8) | 801 (82.7) | 615 (63.4) | 511 (52.7) | 672 (69.3) | 841 (86.8) | 799 (82.4) | 714 (73.7) | 743 (76.7) | 757 (78.1) |
| Yes | 37 (38.1) | 59 (60.8) | 43 (44.3) | 25 (26.0) | 50 (52.1) | 66 (68.0) | 74 (77.1) | 60 (62.5) | 49 (50.5) | 53 (55.2) |
| **Had financial trouble because of someone else's drinking** |  |  |  |  |  |  |  |  |  |  |
| No | 633 (63.1) | 824 (82.2) | 634 (63.2) | 515 (51.3) | 695 (69.3) | 861 (85.8) | 822 (82.0) | 738 (73.7) | 760 (75.8) | 774 (77.2) |
| Yes | 22 (34.9) | 37 (57.8) | 24 (37.5) | 21 (32.8) | 28 (43.8) | 46 (73.0) | 51 (81.0) | 36 (57.1) | 32 (50.8) | 37 (57.8) |
| **Had property vandalised by someone who had been drinking** |  |  |  |  |  |  |  |  |  |  |
| No | 624 (62.8) | 810 (81.7) | 622 (62.6) | 510 (51.4) | 676 (68.1) | 854 (86.0) | 814 (82.0) | 725 (73.0) | 749 (75.5) | 767 (77.2) |
| Yes | 31 (42.5) | 50 (68.5) | 35 (47.9) | 26 (35.6) | 47 (64.4) | 54 (74.0) | 59 (80.8) | 50 (68.5) | 43 (58.9) | 44 (60.3) |

Displayed frequencies and percentages (in parentheses) are weighted.

**Table S3.** *Percentage of survey respondents who had experienced harms due to someone else’s drinking in relation to sex, age and alcohol consumption patterns*

| **Variable** | **Family problems or relationship difficulties due to someone else's drinking** | **Been a passenger with a driver who had too much to drink** | **Been hit or assaulted by someone who had been drinking** | **Had financial trouble because of someone else's drinking** | **Had property vandalised by someone who had been drinking** |
| --- | --- | --- | --- | --- | --- |
| **Sex** |  |  |  |  |  |
| Male | 80 (15.5) | 54 (10.5) | 55 (10.7) | 31 (6.0) | 42 (8.1) |
| Female | 90 (16.4) | 25 (4.5) | 42 (7.6) | 33 (6.0) | 31 (5.6) |
| **Age category** |  |  |  |  |  |
| 18–24 | 22 (17.9) | 12 (9.7) | 15 (12.1) | 8 (6.5) | 9 (7.3) |
| 25–34 | 39 (24.7) | 20 (12.6) | 21 (13.2) | 9 (5.7) | 18 (11.4) |
| 35–44 | 28 (13.8) | 12 (5.9) | 20 (9.9) | 11 (5.4) | 18 (8.9) |
| 45–54 | 39 (21.0) | 20 (10.8) | 25 (13.4) | 19 (10.2) | 17 (9.1) |
| 55–64 | 23 (15.1) | 10 (6.6) | 12 (7.8) | 10 (6.6) | 8 (5.3) |
| 65+ | 19 (7.9) | 6 (2.5) | 5 (2.1) | 5 (2.1) | 3 (1.2) |
| **AUDIT-C** |  |  |  |  |  |
| Non-hazardous drinking | 55 (10.7) | 15 (2.9) | 23 (4.5) | 21.4 (4.1) | 22 (4.3) |
| Hazardous drinking | 115 (20.9) | 64 (11.6) | 74 (13.4) | 42 (7.6) | 51 (9.3) |
| **AUDIT-10** |  |  |  |  |  |
| Low risk | 103 (12.1) | 34 (4.0) | 42 (5.0) | 32 (3.8) | 39 (4.6) |
| Increased risk | 35 (20.5) | 21 (12.3) | 27 (15.8) | 11 (6.5) | 18 (10.5) |
| High risk | 11 (61.1) | 6 (35.3) | 7 (41.2) | 5 (29.4) | 3 (17.6) |
| Alcohol dependent | 22 (81.5) | 19 (70.4) | 21 (77.8) | 15 (57.7) | 13 (50.0) |
| **Binge drinking** |  |  |  |  |  |
| Never | 31 (11.2) | 10 (3.6) | 10 (3.6) | 10 (3.6) | 11 (4.0) |
| Less than monthly | 34 (14.8) | 16 (7.0) | 17 (7.4) | 9 (3.9) | 18 (7.8) |
| Monthly | 17 (15.5) | 6 (5.5) | 9 (8.2) | 3 (2.7) | 10 (9.0) |
| Weekly | 46 (32.4) | 26 (18.2) | 37 (26.1) | 22 (15.5) | 17 (11.9) |
| Daily/almost daily | 10 (40.0) | 10 (40.0) | 11 (44.0) | 8 (32.0) | 8 (32.0) |
| **Drinking to intoxication** |  |  |  |  |  |
| Less than weekly | 100 (14.4) | 43 (6.2) | 50 (7.2) | 31 (4.5) | 46 (6.6) |
| Weekly | 40 (41.7) | 28 (29.2) | 35 (36.5) | 22 (23.2) | 18 (18.8) |

Displayed frequencies and percentages (in parentheses) are weighted.

**Additional references**

41. Clarke N, Pechey E, Mantzari E, et al. Impact of health warning labels communicating the risk of cancer on alcohol selection: An online experimental study. Addiction. 2021; 116(1).

42. Critchlow N, Moodie C, Jones D. Health information and warnings on alcohol packaging in Ireland: It is time to progress the Public Health (Alcohol) Act 2018. Letter to the Editor. Irish Journal of Medical Science. 2022; 191.

43. Hemström O. Attitudes toward alcohol policy in six EU countries. Contemporary Drug Problems. 2002; 29(3).

44. Maani Hessari N, Petticrew M. What does the alcohol industry mean by ‘Responsible drinking’? A comparative analysis. Journal of Public Health. 2017; 40(1).

45. Greenfield TK, Karriker-Jaffe KJ, Giesbrecht N, Kerr WC, Ye Y, Bond J. Second-hand drinking may increase support for alcohol policies: New results from the 2010 National Alcohol Survey. Drug and Alcohol Review. 2014; 33(3).

46. Mercille J. Media coverage of alcohol issues: A critical political economy framework – A case study from Ireland. Int J Environ Res and Public Health. 2017; 14.

47. Manuel T and Davey L. Strategic frame analysis: Providing the ‘evidence’ for evidence-based communications. New Dir Youth Dev. 2009; 124.

48. Tourangeau R, Yan T. Sensitive questions in surveys. Psychological Bulletin. 2007; 133(5).
